# Advice on comparing two independent samples of circular data in biology

**DOI:** 10.1038/s41598-021-99299-5

**Published:** 2021-10-13

**Authors:** Lukas Landler, Graeme D. Ruxton, E. Pascal Malkemper

**Affiliations:** 1 Institute of Zoology, University of Natural Resources and Life Sciences (BOKU), Gregor-Mendel-Straße 33/I, 1180 Vienna, Austria; 2 School of Biology, University of St Andrews, St Andrews, KY16 9TH UK; 3 Max Planck Research Group Neurobiology of Magnetoreception, Center of Advanced European Studies and Research (Caesar), Ludwig-Erhard-Allee 2, 53175 Bonn, Germany

**Keywords:** Animal migration, Behavioural ecology

## Abstract

Many biological variables are recorded on a circular scale and therefore need different statistical treatment. A common question that is asked of such circular data involves comparison between two groups: Are the populations from which the two samples are drawn differently distributed around the circle? We compared 18 tests for such situations (by simulation) in terms of both abilities to control Type-I error rate near the nominal value, and statistical power. We found that only eight tests offered good control of Type-I error in all our simulated situations. Of these eight, we were able to identify the Watson’s U^2^ test and a MANOVA approach, based on trigonometric functions of the data, as offering the best power in the overwhelming majority of our test circumstances. There was often little to choose between these tests in terms of power, and no situation where either of the remaining six tests offered substantially better power than either of these. Hence, we recommend the routine use of either Watson’s U^2^ test or MANOVA approach when comparing two samples of circular data.

## Introduction

Many variables in biology are circular, i.e. they do not fall on a linear scale. Instead, they are recorded on a cyclical scale, for example time of day or compass directions. Such data cannot be analyzed using standard linear statistics; therefore, circular statistical methods have been formulized to detect non-random patterns in periodically recorded data (for discussions on circular data see for example Jammalamadaka and Sengupta^1^, Mardia and Jupp^2^, Mello^3^ and Pewsey et al.^4^). One common example for the use of circular statistics is in orientation biology (see many examples in Batschelet^5^). In a classic experiment, researchers would release pigeons after translocating them to a new location and test if they fly towards home (e.g. they record a vanishing bearing). To test if pigeons orient towards home, one could employ a V-test, which is by far the most powerful test in such a case where a certain heading direction (i.e. towards home) is expected^6^. If the expected heading direction was unknown, a Rayleigh-test would be the most powerful alternative test of the null hypothesis of uniformity of chosen directions. A confidence interval around the mean direction can then help explore if pigeons are significantly oriented towards the home loft or deflected to a different direction.

However, in many scenarios experimenters are not only interested in testing whether a single group shows a significant direction of departure, but they want to compare animals of different origin or test the effect of experimental manipulations, e.g. to understand the underlying orientation mechanisms or cognitive abilities. Such experiments require statistical comparisons between at least two groups, e.g. one group without manipulations (the control) and one with perturbations (the experimental group). Here, we explore the robustness and power of tests designed for the comparison of two groups, which is one of the most common situations, analogous to the use of a two-sample Student t-test or Mann–Whitney-U-test in linear statistics.

Arguably, the most commonly-used test for differences between two circular distributions is the Watson’s U^2^ test^7^, a non-parametric rank-based test that might be thought of as a circular analogue to the Mann–Whitney-U-test. It isimplemented in most software applications that contain circular statistic functions (e.g., Oriana^8^, MATLAB^9^ and *R*^10^). To our knowledge it has never been tested, however, whether the Watson’s U^2^ test is the most robust and powerful test across different situations. To address this, we identified 18 two-sample tests, which can test for similar null hypotheses (i.e. two circular distributions are the same). The mathematical methods underlying the different approaches vary considerably, from classical rank-sum approaches to linear statistics making use of trigonometrical functions. For brevity, we only considered test versions where the p-value is derived asymptotically rather than by randomization/bootstrapping. Our aim was to provide advice on which tests to use in which situations.

We grouped the type of comparisons between two distributions according to three possible null hypotheses: (1) The distributions are identical, (2) they have the same mean/median, or (3) they show the same concentration. Type (1) tests should be sensitive to differences in mean/median as well as to differences in the concentration, however. Type (2) tests should be sensitive to differences in the mean/median, but not show differences when only the concentration varies. In contrast, type (3) tests should be sensitive to differences in concentration but not detect differing means/medians. None of the tests should be sensitive to differences in sample sizes.

Another level of complexity in choosing a method is that certain tests require defined prerequisites to show reliable results. The most common prerequisite is that distributions should be oriented unimodally (see for example the Watson–Williams test) or that the sample size should exceed a minimum size (e.g. this is true for the Watson’s large-sample nonparametric test)^4^. It is unclear how robust the tests are against violation of the assumptions, but our study will offer guidance on this.

In summary, in this simulation study we explore a myriad of available tests in the most common situations where two independent circular distributions are compared and subsequently derive recommendations for scientists working with circular data. Our analysis includes unconventional linear approaches, as well as predominantly used circular statistical tests.

## Methods

### Statistical tests used in simulation

In total we used 18 tests implemented in several *R* packages (Directional^11^, CircStats^12^, circular^13^, kuiper.2samp^14^, lawstat^15^, NPCirc^16^ and Rfast2^17^), code for *R* functions provided in Pewsey et al.^4^, or, in the case of the P-test, newly implemented in R for this manuscript (see Table 1, Supplementary Material for details on functions and packages used). The P-test followed the calculations provided in Rumcheva and Presnell^18^.

**Table 1:** ﻿List of tests used, including the functions and packages used, as well as the respective null hypothesis and test abbreviation.

Test name	Abbreviation	Function	Package/resource	Test assumptions
**Identical distribution**				
Embedding approach ANOVA	EmA	embed.circaov	Rfast2	None given
Kuiper two sample test	Kui	kuiper.2samp	kuiper.2samp	None given
Large-sample Mardia-Watson-Wheeler test	MWW	WgVal	Pewsey et al. 2013	n for each sample > 10
Log-likelihood ratio ANOVA	LlA	lr.circaov	Rfast2	None given
MANOVA approach	Man	manova	stats	None given
Non equal concentration parameters approach ANOVA	HeA	het.circaov	Rfast2	None given
Watson–Wheeler test	WWe	watson.wheeler.test	Circular	Continuous data (ungrouped)
Watson’s U^2^ test	WU2	watson.two.test	Circular	Combined sample size > 17
**Identical mean/median**				
Fisher’s nonparametric test	FPg	PgVal	Pewsey et al. (2013)	None given
P-test	Pt	P.test.assym	R-code provided in this paper	None given
Rao polar test	Rpo	rao.homogeneity	CircStats	Large unimodal samples, not polar opposite
Watson–Williams test	WWi	watson.williams.test	Circular	von Mises, same concentration (k > 2)
Watson’s large-sample nonparametric test	Wat	YgVal	Pewsey et al. (2013)	None given
**Identical concentration**				
Concentration test	Con	conc.test	Directional	None given
Fisher’s method	FM	tang.conc	Directional	None given
Levene’s test	Lev	levene.test	lawstat	None given
Rao dispersion test	Rdi	rao.homogeneity	CircStats	None given
Wallraff test	Wal	wallraff.test	Circular	Continuous data (ungrouped)

### Simulated distribution samples

**Figure 1:**
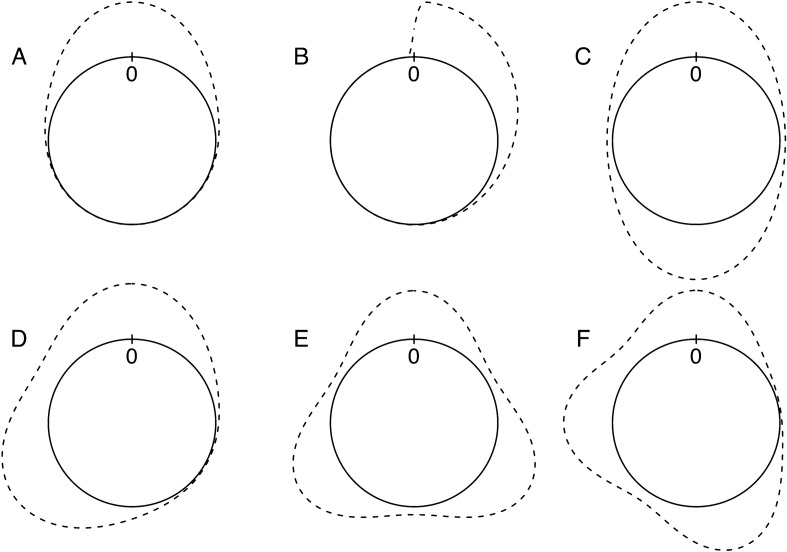
Densities for distribution types used in this paper: unimodal von Mises (**A**), unimodal wrapped skew-normal (**B**), axial von Mises (**C**), asymmetrical bimodal von Mises (**D**), symmetrical trimodal von Mises (**E**), asymmetrical trimodal von Mises (**F**).

All samples were generated using the *rcircmix* function from the NPCirc package (R code in Supplementary Material, see Fig. 1 for distributions used), using “vm” and “wsn” distribution types for von Mises and wrapped skew-normal, respectively. Each simulation was performed with 9999 randomly drawn samples from the given distribution, the proportion of significant test results is then reported as Type-I error or power. For all comparisons, we used the same sample size pairs per mode. For unimodal distributions (e.g. Fig. 1A,B) we used sample sizes of 10/10 (sample size for sample 1/ sample size for sample 2), 20/20, 50/50, 20/30, 10/50; for bimodal (axial: 0° and − 180° (e.g. Fig. 1C), non-symmetrical: 0° and − 120° (e.g. Fig. 1D)) and trimodal (symmetrical: 0°, − 120° and − 240° (e.g. Fig. 1E), non-symmetrical: 0°, − 90° and − 200° (e.g. Fig. 1F)) distributions we doubled and tripled the sample sizes, respectively.

### Type-I error

For each of the distributions we evaluated the Type-I error probability by comparing two identical distributions, the concentration/dispersion parameters were increased for both. We only continued evaluating the power of those tests that were robust, i.e. that maintained a Type-I error level of around 5% in all situations. (Note: The power of all tests was calculated and can be found in the Supplementary Results sheets, however, it was not included in the figures for clarity).

### Power calculations—difference in concentration

In order to evaluate the power of the tests to detect differences in concentrations, we varied the concentration parameters of one of the distributions and kept the second one constant. For the von Mises distribution we used the concentration parameter κ = 0 (i.e., a uniform distribution) for one and values from 0 to 8 for the other distribution. For the wrapped skew-normal, one distribution was kept at the dispersion parameter ρ = 1 and for the second it ranged from 1 to 4 (in all cases a skew parameter of 30 was used).

### Power calculations—difference in mean/median

To evaluate the power to detect differences in directionality of unimodal distributions, we kept one of the two distributions fixed at a mean of 0° and used mean directions from 0° to 180° for the second one. For bimodal distributions, we used directions from 0° to 90° and for trimodal distributions, we used 0° to 60° in order to encompass the range where we expected most differences between distributions.

### Power calculations—difference in distribution type

To test the power of the tests to differentiate between different types of distributions, we compared two distributions with similarly increasing concentration parameters across simulations (dispersion parameter in the case of the wrapped skew-normal distribution), while keeping the directionality of the distributions the same between the distributions. For all the analyses, we kept the sample sizes the same as before for the respective distributions. First, we compared a unimodal von Mises distribution with a symmetrical bimodal (= axial) von Mises distribution, both aligned on the same axis. Again, the concentration parameter increased for both in the same manner. In a second simulation, we compared unimodal von Mises with unimodal wrapped skew-normal distributions, using the same parameters as for the Type-I error calculations, for both (only the dispersion parameter range was flipped to range from 4 to 1 for the wrapped skew-normal distribution, to follow the concentration parameter better).

### Real data examples

To test the performance of the tests in real life situations, we applied the identified robust tests to three published examples from biological studies. The first data set (*pigeon*) is included in the *R* circular package and describes a translocation experiment with homing pigeons^19^, where the performance of control animals was compared to pigeons with a sectioned olfactory nerve (note: a third group present in the original paper was not included in our analysis). It was expected that the control group is oriented (highly clustered) and the experimental group is disoriented (not clustered), causing a difference of concentration between the groups.

The second data set is from work by Wehner and Müller^20^ that investigated the ability of desert ants (*Cataglyphis fortis*) to transfer directional visual information received in one eye, to the other eye without further training (data included in the *R* circular package: *fisherB10c*). For the purpose of this analysis, we compared a control group (trained and tested with the same eye open) with a treatment group (trained with one eye and tested with the other eye). The hypothesis here was that information transfer between the two visual views is possible. The expectation, therefore, is a lack of difference between groups, both in direction and concentration.

The third data set is taken from a recent paper on bat navigation by Lindecke et al.^21^. They investigated if migratory bats (*Pipistrellus pygmaeus*) use the sun’s position at dusk to calibrate their navigational compass. For our example, we only analyzed the data from adult animals. Here the expectation was that the recorded headings should not differ in concentration, but show opposite directional preferences, according to whether the bats saw natural or mirrored views of the sun.

## Results

### Type-I error

**Figure 2:**
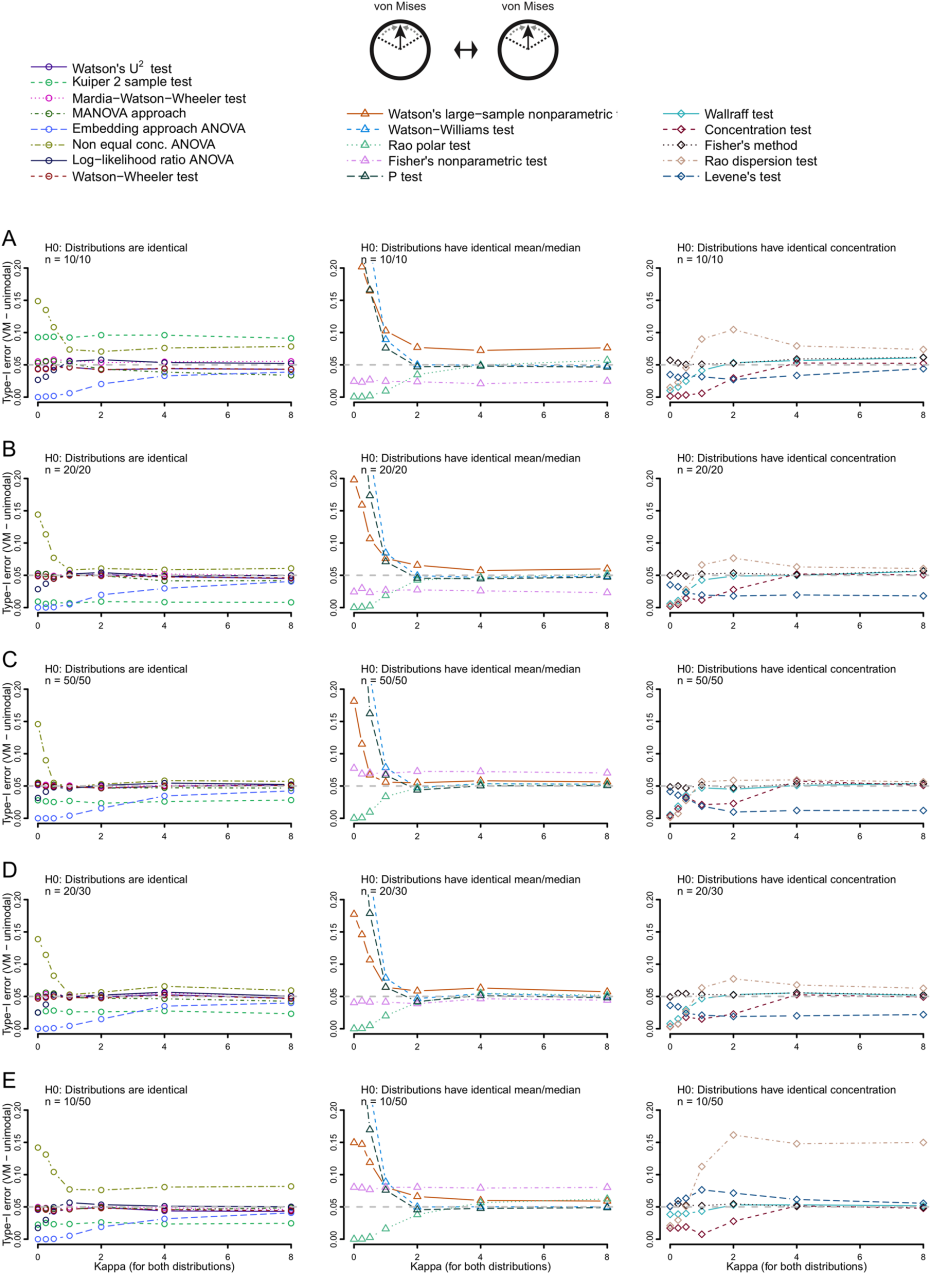
Type-I error of all tests using von Mises distributions for different sample sizes: 10 and 10 (**A**), 20 and 20 (**B**), 50 and 50 (**C**), 20 and 30 (**D**) and 10 and 50 (**E**). Concentration (κ, kappa) increases for both distributions from 0 to 8. Tests are grouped according to their null hypotheses.

In the case of two identical unimodal von Mises distributions, seven tests did not maintain Type-I error near the nominal 5% level, at least when sample sizes were small. These tests were the Kuiper two-sample test, the non equal concentration parameters approach ANOVA, the P-test, the Watson’s large-sample nonparametric test, the Watson–Williams test and the Rao dispersion test (Fig. 2). The Type-I error results were similar for the unimodal wrapped skew-normal distribution, except that the Wallraff test and Fisher’s method also showed Type-I error inflation (Fig. S1). No other methods showed evidence of failure to control Type-I error rate across different testing situations (Figs. S2–S5), except for the Log-likelihood ratio ANOVA in the case of two identical asymmetrical bimodal distributions (Fig. S3). In summary, only eight out of 18 tests reliably controlled the Type-I error rate near the nominal 5% level across all the situations investigated. These included five tests for identical distribution, the Watson’s U^2^ test, the Large-sample Mardia–Watson–Wheeler test, the Watson-Wheeler test, the embedding approach ANOVA, the MANOVA approach, the Rao polar test for differences in mean direction, and two tests for differences in concentration, the Levene’s test and the concentration test. We focus only on these tests in our explorations of statistical power.

### Power to detect differences in concentration

**Figure 3:**
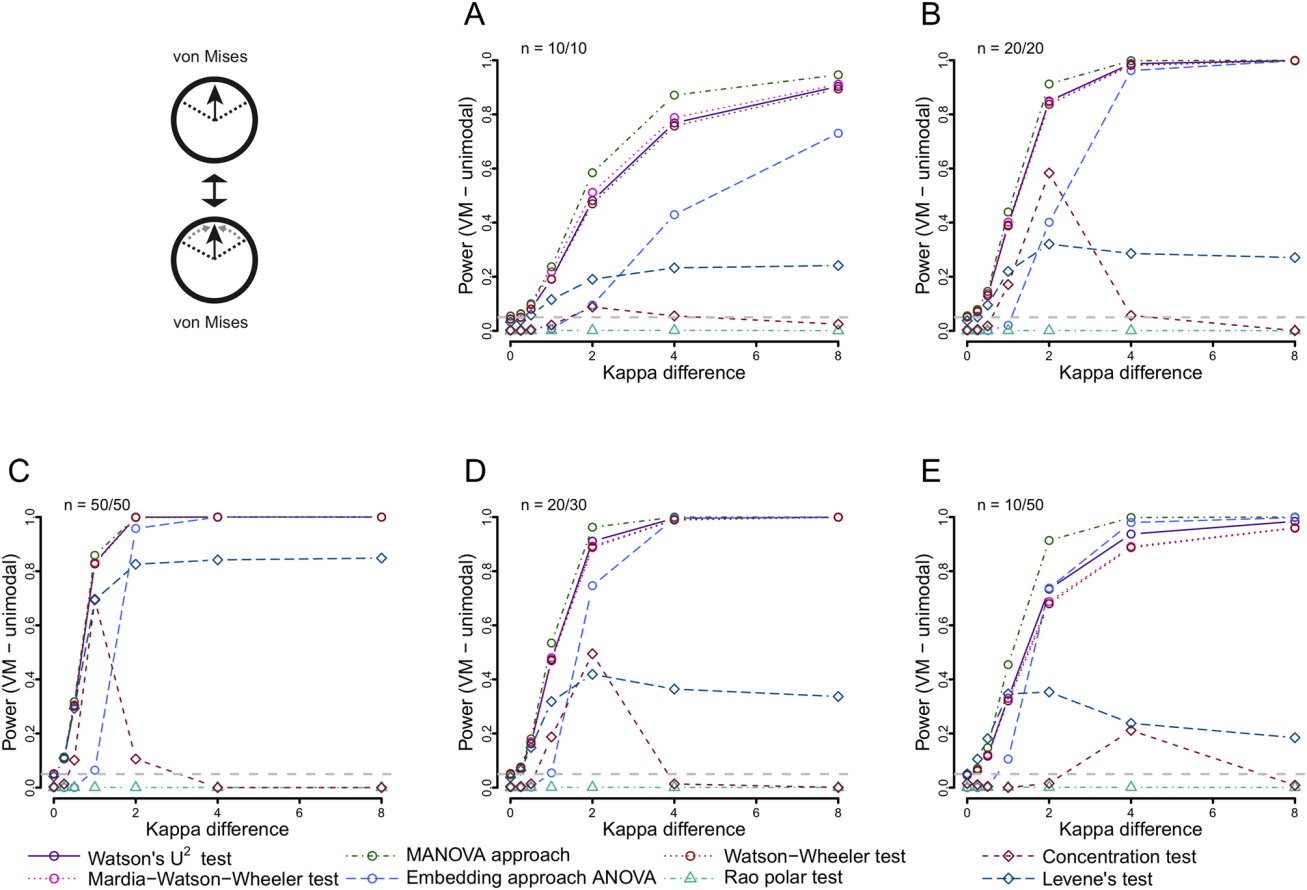
Power of all included tests when comparing von Mises distributions of differing concentrations using different sample sizes: 10 and 10 (**A**), 20 and 20 (**B**), 50 and 50 (**C**), 20 and 30 (**D**) and 10 and 50 (**E**). The first distribution is fixed at κ = 0, the second increases from 0 towards 8.

The most powerful test to detect concentration differences between two von Mises distributions was the MANOVA approach, which offered superior power especially at lower sample sizes (Fig. 3). The Watson’s U^2^ test was also very powerful, followed by the Watson–Wheeler and the Large-sample Mardia–Watson–Wheeler tests with only marginally lower power. The embedding approach ANOVA had lower power, but, notably, was still more powerful than the Concentration test and Levene’s test, both specifically designed to detect differences in concentration. As expected, the Rao polar test was not sensitive to differences in concentration. The general results for two unimodal wrapped skew-normal distributions were comparable to the results for unimodal von Mises distributions, with the only exception of superior performance of Levene’s test in situations with highly asymmetric samples sizes (Fig. S6).

When comparing axial von Mises distributions, only the Watson’s U^2^ test offered acceptable power (Fig. S7). For the symmetrical trimodal distributions, overall power was very low, and again, only the Watson’s U^2^ providing some power (Fig. S8). The asymmetrical bimodal (Fig. S9) situation showed acceptable power of the MANOVA approach and Watson’s U^2^, however, for the asymmetrical trimodal distribution power was low with the Watson’s U^2^ providing the best results (Fig. S10).

### Power to detect differences in the mean/median

**Figure 4:**
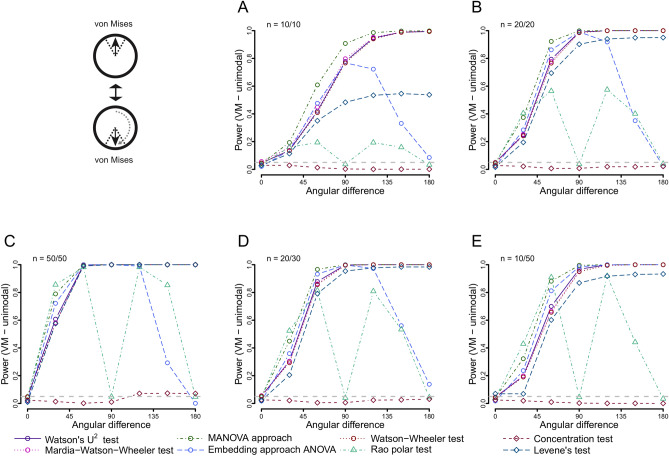
P﻿ower of all included tests when comparing von Mises distributions (kappa for both = 2) of differing directions using different sample sizes: 10 and 10 (**A**), 20 and 20 (**B**), 50 and 50 (**C**), 20 and 30 (**D**) and 10 and 50 (**E**). The first distribution is fixed at 0°, the second increases from 0° towards 180.

The power to detect angular differences between two von Mises distributions was highest for the MANOVA approach at small sample sizes (n = 10), followed by the Watson’s U^2^, Watson-Wheeler test and the Large-sample Mardia-Watson-Wheeler test (Fig. 4). Notably, the Levene’s test also showed acceptable power levels, clearly failing to detect specifically concentration differences (to which it was less sensitive, see Fig. 4). The concentration test was not sensitive to the differences in mean direction. Special cases were the embedding approach ANOVA and the Rao polar test. The ANOVA approach showed, with the exception of very unequal sample sizes (n = 10/50), a unimodal response, with increasing power levels from 0° to 90° difference, but then rapidly decreasing power towards 180° difference. The Rao polar test showed an even stranger pattern, with, at higher sample sizes, very good power when the difference was either around 45° or 135°, but with power levels dropping to 0.05 in between these two peaks (at 90°). The results were similar for the wrapped skew-normal distribution, with the exceptions that the Rao polar test showed strongly reduced power and switched from a bimodal to a unimodal power curve with a peak around 60°, and the Levene’s test completely lost its power (Fig. S11).

For axial distributions, only the Watson’s U^2^ test offered acceptable power levels, although large sample sizes (~ n = 100) were required for the power to reach over 50% (Fig. S12). All other tests failed to detect the difference in mean direction between two axial distributions. For symmetric trimodal distributions none of the tests used was sensitive to differences in mean direction (Fig. S13).

When comparing asymmetrical bimodal distributions, the general trends were similar to the unimodal case. However, over all sample sizes the MANOVA approach offered the best power. The Watson–Wheeler test was considerably less powerful in this situation, as were the Watson’s U^2^ test and the Large-sample Mardia–Watson–Wheeler test (Fig. S14). The Levene’s test showed a unimodal-shaped power curve. The asymmetrical trimodal situation was, again, similar to the asymmetrical bimodal situation (Fig. S15), with the exception of the Levene’s test, which showed steady power increase with angular difference (instead of the hump-shaped curve).

### Power to detect differences in distribution type

**Figure 5:**
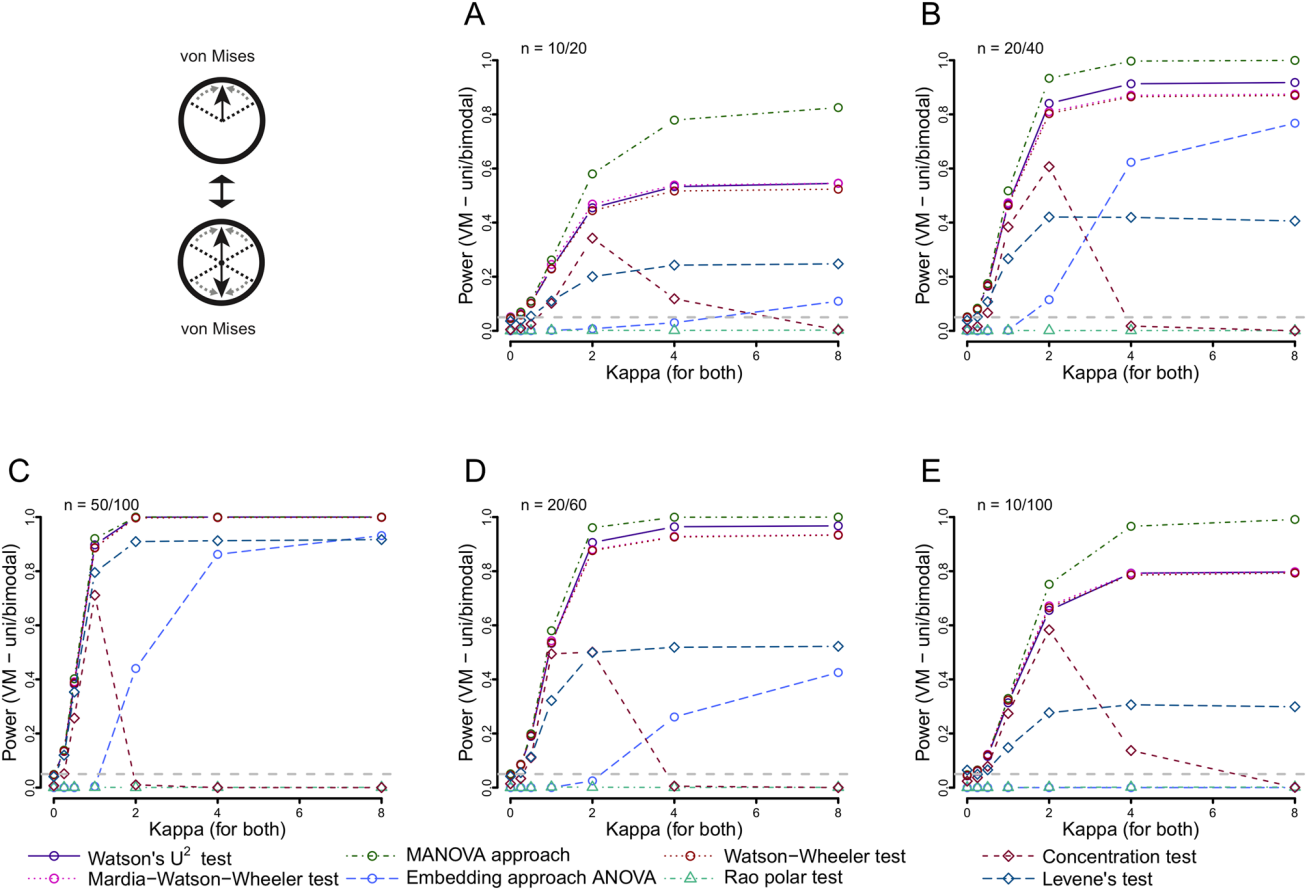
Power of all included tests when comparing von Mises distributions of differing number of modes (unimodal and axially bimodal) using different sample sizes: 10 and 20 (**A**), 20 and 40 (**B**), 50 and 100 (**C**), 20 and 60 (**D**) and 10 and 100 (**E**). The concentration (κ) of both increases from 0 to 8.

When comparing a unimodal and an axial bimodal distribution, which increased similarly in concentration, we found that the MANOVA approach again offered the best power in particular at low samples sizes, followed by the Watson’s U^2^ test, the Large-sample Mardia–Watson–Wheeler test and Watson–Wheeler test (Fig. 5). While the embedding approach ANOVA and the Levene’s test had varying but usable power levels, the concentration test was only sensitive to such differences at low concentration values. The Rao polar test was not sensitive to such differences.

The picture was only marginally different when comparing a von Mises with a wrapped skew-normal distribution (Fig. S16). For low sample sizes (n = 10) the MANOVA approach offered great power, followed by the embedding approach ANOVA. The latter offered good power throughout the range of sample sizes tested, followed by the Watson’s U^2^ test, the Large-sample Mardia–Watson–Wheeler test and Levene’s test. Also, the Rao polar test showed lower, but acceptable sensitivity to distribution type. The concentration test only showed very low power, that (as expected) increased with increasing concentrations of the respective distributions.

We summarize the results obtained in the power analysis in Table 2. In all situations, either the Watson’s U^2^ test or the MANOVA approach offered the best power.

**Table 2:** Ranking of tests based on the power comparisons for the main scenarios encountered in potential data sets (using different distributions: unimodal, axial, asymmetrical bimodal, symmetrical trimodal, asymmetrical trimodal), in cases were only one test performed acceptable the others ranks were left blank (see Table 1 for abbreviations).

Scenario	Unimodal	Axial	Asym. bim	Sym. trim	Asym. trim	Different dist. types
Rank 1	Man	WU2	Man	WU2	WU2	Man
Rank 2	WU2		WU2		Man	WU2
Rank 3	MWW		MWW		MWW	MWW
Rank 4	WWe		WWe		WWe	WWe

### Real data examples

**Figure 6:**
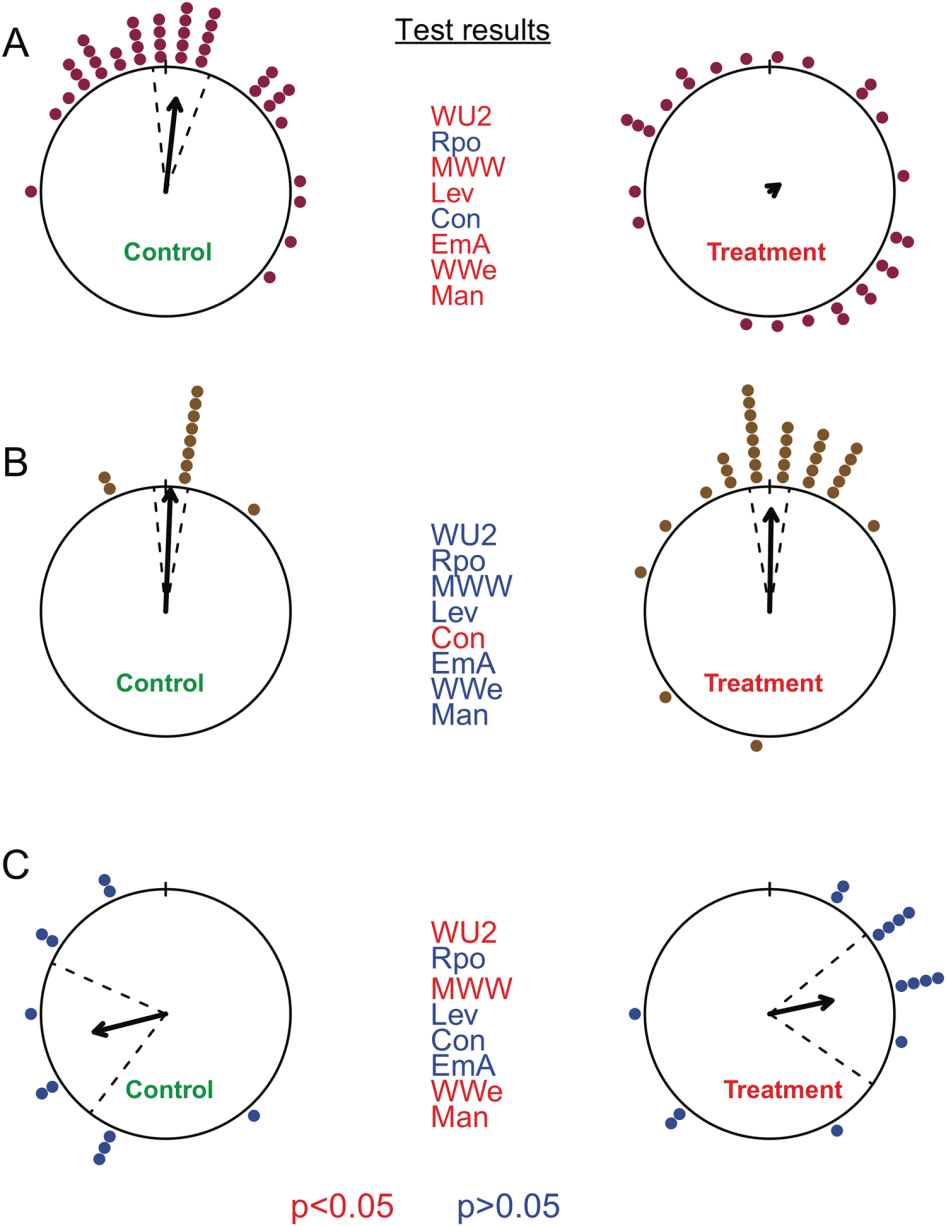
﻿Results from example data. Shown are results of pigeon (**A**), ant (**B**) and bat orientations (**C**). Control groups are on the left panels and experimental groups on the right. The tests are abbreviated according to Table 1, significant test results are indicated in red with asterisk and non-significant in blue. For each circular plot directional data is shown as dots on the circle (each dot is one individual), the arrows represent the mean direction and the dashed line the 95% confidence interval.

Testing the performance of the robust tests on real data sets revealed, predominantly, the expected test behavior. In the example of homing pigeons where a difference in concentration was expected, all tests, with the exception of the Rao polar test and, notably, the concentration test, showed a significant difference between the distributions (Fig. 6A). Therefore, we can conclude, in accordance with the respective publication^19^, that sectioning of the olfactory nerve disrupted the homing behavior of pigeons.

In the ant example, where no difference between the groups was expected, there was no significant difference between the distributions detected by most of the tests (Fig. 6B). Only the concentration test showed a significant difference. Based on the other tests we would conclude that there was no biological meaningful difference between the two distributions. Therefore, ants appear to be able to transfer visual information from one eye to the other.

In the bat example, where a difference in mean direction was expected, the Watson’s U^2^, the Mardia–Watson–Wheeler, Watson-Wheeler test and the MANOVA approach showed a significant difference (Fig. 6C). Notably, the Rao polar, Levene’s, and concentration tests and the embedding approach ANOVA failed to show a significant difference. At least for the Rao polar test, one would have expected a significant difference, as the two distributions are clearly 180° apart. This outcome concurs with our simulation results where the Rao polar test failed to distinguish distributions on the same and orthogonal axes (Fig. 4). As the results of the tests where quite mixed this example highlights the need for choosing a test with appropriate power to detect the expected differences. Based on the results of the most powerful tests, we conclude that the bats showed a mirrored orientation, as expected in the experimental design.

## Discussion

Our extensive power analyses of tests for analyzing if two circular distributions are the same show that many of the tests suffer from elevated Type-I error rates, at least in specific situations. To minimize the risk of getting false positive results we recommend not using any of these tests. Of the eight remaining tests, only one test, the Watson’s U^2^ test, offers good power in all of the tested situations. An interesting alternative revealed by our analysis, however, is the MANOVA approach, which is also very powerful, and furthermore allows the addition of covariates.

To our knowledge, the MANOVA approach, as used in this analysis, has only been used once in similar fashion in order to test the effect of environmental factors on toad orientation^22^. Nobody seems to have used it for distribution comparisons. This is surprising, as the underlying ideas (transforming an angle in two linear components using trigonometric functions) and the MANOVA calculation itself are not at all new to statistics. Given the potential advantages of such an approach in terms of adding covariates and its robust and powerful behavior we would recommend the broader uptake of this test in circular statistics when it comes to group comparisons.

Some of the tests that we did not evaluate further might be very powerful for a certain set of situations, where they maintain expected Type-I error rates. However, such utilization of statistical tests can easily lead to false positive results and in general might be quite delicate to handle for the experimenter. They would have to inspect their distributions closely before testing. Such test selection on the basis of visual inspection of the data by itself could lead to increased Type-I error rates. In contrast to linear statistics where certain distributions can be expected by study design (e.g. random sampling of continuous variable would suggest a Gaussian distribution, or count data a Poisson distribution), this is not the case for circular statistics. In addition, it is important to note that permutation-based tests were not evaluated in this paper, but these could control Type-I error and may even outperform traditional tests (see Noguchi et al.^23^ for permutation-test performance for linear data).

It should also be added that a Bayesian circular GLM approach, which can also include random effects, was introduced by Cremers and Klugkist^24^. Comparison of the MANOVA approach with such a Bayesian alternative would certainly be a fruitful avenue of future research, to expand the circular statistics toolbox further.

The behavior of the concentration test is puzzling; in general, it has low power to detect differences, but appears to be highly sensitive to very specific differences. From inspection of the results of the ant example (see Fig. 6B) it is clear that the two distributions are not identical in terms of clustering, although the confidence intervals and mean vector lengths are similar. In the control group most animals are exactly at the same direction, while there is a more even spread in the experimental distribution. Therefore, the detected difference might be real, but it is unclear what feature of the data exactly this test detects.

In case of expected axial orientation for both distribution we recommend doubling the angles and reducing to modulo 360° for both distributions prior to applying any of the tests, in order to overcome the extreme loss of statistical power in such situations (e.g. Fig. S7). If this is not possible or attractive to the researcher, the best option is using the Watson’s U^2^ test.

In the current study, we only tested two distributions against each other, although six out of the eight tests included in the power analysis would also allow testing of multiple distributions. We surmise that the general power comparisons between those tests would show similar patterns also when considering more groups. Exploring the performance of tests using multiple treatment groups would be another fruitful avenue of research, as would evaluation of paired data.

## Supplementary Information

Supplementary Information 1.

Supplementary Information 2.

Supplementary Information 3.

## Data Availability

Data and code are available in the Supplementary Material.
