# Supplementary material for: Advice on comparing two independent samples of circular data in biology
Source: Sci Rep. 2021 Oct 13;11:20337. doi: 10.1038/s41598-021-99299-5 (PMC8514454; doi:10.1038/s41598-021-99299-5)
Supplement: Supplementary file 3 — Supplementary Information 3. [file 41598_2021_99299_MOESM3_ESM.docx]

**Appendix**

**Comparing two circular distributions: advice for effective implementation of statistical procedures in biology**

Lukas Landler^1*^, Graeme D. Ruxton^2^, E. Pascal Malkemper^3^

*corresponding author

[lukas.landler@boku.ac.at](mailto:lukas.landler@boku.ac.at)

We assume that we have two samples of angles in radian measure (between 0 and 2π) of sample sizes *n* and *m*: sample *A* has values *α_1_*, ……, *α_n_* and sample *B* has values *β*_1_, …, *β_m_*.

**Watson-Williams test**

This tests the null hypothesis that the samples are drawn from underlying von Mises distributions with the same mean.

For sample A, we can define

$\begin{matrix} C_{A}=\sum_{i=1}^{n} Cos\left( \alpha_{i} \right) & S_{A}=\sum_{i=1}^{n} Sin\left( \alpha_{j} \right) \end{matrix}$,

And then the resultant length of sample *A*,

$$R_{A}=\sqrt{C_{A}^{2}+S_{A}^{2}}$$

We can similarly obtain the resultant lengths of sample *B* (*R_B_*) and of the combined sample of size *N* (*N*=*n*+*m*): *R*. Now if the underlying populations where the same von Mises distribution then we should expect *R_A_*+*R_B_* = *R*. So for this test we reject the null hypothesis for large enough values of the test statistic:

$$F_{W}=g\left( N-2 \right)\frac{R_{A}+R_{B}-R}{N-R_{A}-R_{B}}$$

Where the constant

$$g=1+\frac{3}{8\kappa}$$

Where *κ* is an estimate of the concentration parameter of the two underlying von Mises distributions. Batchelet offers a table (his table D1) for estimating this on the basis of the mean of the two single sample resultant vectors and the mean of the two sample sizes. To obtain a p-value the test statistic is compared with *F_1,N-2_*.

Assumptions of the test: both underlying populations are von Mises with the same concentration parameter, which is greater than 2.

Remarks: The consequences of deviating from the assumptions of the test are unexplored. It should be noted that the test statistic is only expected to approximately follow an *F* distribution, and the quality of this approximation, likely to be questionable at small sample sizes, is unknown.

The test was introduced by Watson & Williams (1956) with g = 1, the improvement with g >1 is due to Stephens (1972).

**Watson’s large-sample nonparametric test**

This is a non-parametric test of the null hypothesis of a common mean direction: originally due to Watson (1983), it is defined in detail by Fisher (1995) and Pewsey et al. (2013). The mathematics are a little involved (so not repeated here) but essentially rely on estimation of the difference between the means. The derivation makes no assumption that the underlying distributions have a similar shape or concentration. However, it might be argued that testing the null hypothesis of a common mean direction is only really sensible if the underlying distributions are unimodal and symmetric. Pewsey et al. (2013) offer a randomization version for situations where either *m* or *n* is less than 25, and an asymptotic test based on chi-squared otherwise. The test does not seem to have been subject to extensive testing of its properties.

**Fisher's nonparametric test**

This is described in Fisher (1995) and Pewsey et al. (2013). It tests the null hypothesis of a common media direction to the underlying distributions. There is no assumption that the underlying distributions are the same, are symmetric, or have similar concentrations. To apply this test, each data-point is replaced by its difference from the common median angle calculated across both samples. These differences are then scaled to lie between -π and π, and the numbers of negative values in each sample calculated – as *n_a_* and *n_b_*. The test value is then

$$P_{g}=\left( \frac{N^{2}}{\left( n_{a}+n_{b} \right)\left( N-n_{a}-n_{b} \right)} \right)\left( \frac{n_{a}^{2}}{n}+\frac{n_{b}^{2}}{m}-\frac{N\left( n_{a}+n_{b} \right)}{N-n_{a}-n_{b}} \right)$$

If both samples sizes are ten or above then this can be compared to a chi-squared distribution with one degree of freedom; otherwise a randomization approach is taken. This method has not been subject to extensive evaluation.

**Watson-Wheeler test**

A non-parametric test that tests the null hypothesis that the two underlying populations are identical. Any difference between the populations can trigger rejection of the null hypothesis. The only restriction on the use of this test is that the data should be continuously distributed (ungrouped) so that there are no tied values, but even this can be circumvented.

To apply the test we merge the two samples and rank all *N* values, keeping the identity (either *A* or *B*) of each ranked value. Imagine that sample *A* contributes the lowest value and the 5^th^ lowest value, then we replace the two associated values in sample *A* with (2π)/*N* and 5(2π)/*N* respectively. We carry on this way until we have replaced all the values in both samples with these standardised ranked values. We then calculate the resultant lengths *R_A_* and *R_B_* in exactly the same way as for the Watson-Williams test. If the two populations are the same then both samples will be uniformly distributed around the circle and both resultant vectors will be zero. Thus large values for these resultant vectors argues for rejection of the null hypothesis. The test statistic (*B*) is simply the square of larger sample resultant length of the first sample (*R_A_* or *R_B_* in our case). This is compared either with a critical value from a table (Table Q in Batschelet (1981) for *N* < 18). For N > 17, then the quantity

$$\frac{2\left( N-1 \right)B}{nm}$$

has approximately a chi-squared distribution with two degrees of freedom.

Notes: This test was proposed originally by Wheeler and Watson (1964). and independently by Mardia (1967). You can also find it called the *Mardia-Watson-Wheeler* test or the *uniform-score* test.

It has been suggested that if ties occur then they could be randomly broken. That is, if we imagine that both samples A and B have the same value and these two values are ranked 2^nd^ and 3^rd^ lowest say, then two ranks are allocated by simulating a coin toss. All ties could be dealt with in this way, but the consequence of this for test performance has not been evaluated, nor has the quality of the chi-squared approximation.

**Large-sample Mardia-Watson-Wheeler test**

This is a very slight variation on the Watson-Wheeler test, and is described by Pewsey et al. It draws more on the paper by Mardia (1967), whereas the implementation in the *Watson.wheeler.test* function in the *circular* package draws more on the Wheeler and Watson (1964) paper. In the Watson-Wheeler test the test statistic with *R_A_* > *R_B_* was

$\frac{\boldsymbol{2}\left( \boldsymbol{N-1} \right)\boldsymbol{R}_{\boldsymbol{A}}^{\boldsymbol{2}}}{\boldsymbol{nm}}$**,**

Now here the test statistic is

$$\boldsymbol{2}\left( \frac{\boldsymbol{R}_{\boldsymbol{a}}^{\boldsymbol{2}}}{\boldsymbol{n}}\boldsymbol{+}\frac{\boldsymbol{R}_{\boldsymbol{B}}^{\boldsymbol{2}}}{\boldsymbol{m}} \right)\boldsymbol{.}$$

Pewsey’s implementation uses the same chi-squared approximation (with two degrees of freedom) if both sample sizes are above ten, and randomisation otherwise.

**Kuiper’s two sample test**

This again is a non-parametric test of the null hypothesis that the two underlying populations are identical. It does not have difficulty with grouped data, but Batschelet (1981) recommends that the class interval be small (not larger than 5 degrees). This test is the application (due to Kuiper (1960)) of the Kolmogorov-Smirnov test to circular data. It involves construction of the cumulative frequency functions from the two samples, call these C_a_ and C_b_. Imagine for convenience that we order the values in sample A and convert them to degrees, and that the first four values are 10^o^, 25^o^, 25^o^ and 30^o^. The *C_a_*(*x*) is a cumulative set of steps from zero at zero degrees up to 1 at 360 degrees. In climbs up to 1/*n* at 10^o^, up to 3/*n* at 25^o^ and 4/*n* at 30^o^ etc. We can define define C_b_(x) similarly. We define D+ as the maximum height that *C_a_* is above *C_b_* at any point, and D- as the maximum height that C_b_ is above C_a_ at any point. The test statistic *k* is then given by

$k=nm\left( D^{+} + D^{-} \right)$,

With critical values being listed in Table S of Batschelet (1981). However the R function *Kuiper.2samp* uses calculated values from Paltani (2004).

**Watson’s *U^2^* test**

This test (due to Watson (1962)) again is a non-parametric test of the null hypothesis that the two underlying populations are identical, and has similarity to Kuiper’s test in being based on the cumulative frequency functions. In the absence of ties there are *N* distinct points at which one or other curves changes, and so *d_1_*,….,*d_N_* points where we can measure a difference in height between the two curves. Treat all these values as positive, and define D as their mean value. Then the test statistic U^2^ is as follows:

$$\boldsymbol{U}^{\boldsymbol{2}}\boldsymbol{=}\left( \frac{\boldsymbol{nm}}{\boldsymbol{N}} \right)\left( \sum_{\boldsymbol{i=1}}^{\boldsymbol{N}} \boldsymbol{d}_{\boldsymbol{i}}^{\boldsymbol{2}}\boldsymbol{-}\frac{\boldsymbol{1}}{\boldsymbol{N}}\left( \sum_{\boldsymbol{i=1}}^{\boldsymbol{N}} \boldsymbol{d}_{\boldsymbol{i}} \right)^{\boldsymbol{2}} \right)$$

The higher the test value the more likely the null hypothesis is to be rejected. Critical values can be found in table T of Batschelet (1981). Watson.2.test in the circular package states: “Critical values for the test statistic are obtained using the asymptotic distribution of the test statistic. It is recommended to use the obtained critical values and ranges for p-values only for combined sample sizes in excess of 17.” This is probably not a large source of error, because the critical values (see table B.38 of Zar (2009)) change relatively little with sample size.

**Wallraff test**

This tests the null hypothesis that two samples are drawn from populations with the same dispersion. First each angle in the sample is converted to an angular distance from a reference direction specific to that sample. In the *Wallraff.test* implementation these reference directions can be specified, but if they are unspecified then the sample mean is used as the reference direction for each sample. Then a Mann-Whitney test (without continuity correction) is applied to the differences. Since this is a rank test, the Wallraff test makes no assumption about the shapes of the distributions, and does not even require the assumption that the two are the same. It similarly makes no assumption about whether the means are the same (provided no difference in reference directions is specified). It does assume that there are no tied values, and the performance of the test in the presence of ties seems unexplored.

**Levene’s test**

Levene (1960) proposed a test for homogeneity of variances in *k* groups that is based on the ANOVA statistic applied to absolute deviations of observations from the corresponding group mean. The robust Brown-Forsythe version of the Levene-type test substitutes the group mean by the group median in the classical Levene statistic, and this has been demonstrated to offer improved robustness to underlying non-normality. Although this test was not specifically designed for circular data, it could be used as an equivalent to the Wallraff test, but using absolute rather than rank deviations. Its performance with respect to circular data has not been explored.

**Fisher’s method**

Fisher (1995) offers a parametric method for testing the null hypothesis that two von Mises distributions have identical concentrations – the method makes no assumption about the means of the distributions being identical. Calculation of the test statistic is described on page 140 of Pewsey et al. (2013), and this is compared with values from *F* tables with 2 and *N*-2 degrees of freedom. This comparison is only recommended if both sample sizes are at least 10, otherwise Pewsey et al. (2013) recommend a randomisation alternative. The *tang.conc* function in R always appears to use to the asymptotic version of the test regardless of sample sizes. The conc.test function implements a different test of the same null hypothesis using theory drawn from ANOVA – detailed on page 133 of Mardia and Jupp (2009).

**P-test**

The most extensive study of tests comparing two circular samples is that of Rumcheva and Presnell (2017). They compare their own novel test (hereafter P-test) with the original Watson-Williams test (W-test) and Stephen’s modification of the Watson-Williams test (M-test). They also consider a test due to Harrison et al. (1986) based on ANOVA (A-test) and a maximum-likelihood test due to Mardia & Jupp (2009) (G-test). They do not consider a test due to Upton (1976) since they suggest that Upton’s results suggest that it is no more powerful than the M-test but controls type one error less well (hereafter, we call the ability to control type I error rate at the specified level as the “accuracy” of the test).

The simulation results of Rumcheva and Presnell (2017) suggest that the P-test is always accurate, the G and A tests show poor accuracy (see their table 1). The M-test shows reasonable accuracy in most situations studied, but is never significantly better than the P-test and is sometimes worse than the W-test. The power of the P-test always stands good comparison with other tests, despite the fact that the others are often anti-conservative. Further, its derivation sets no restriction on the size of the common concentration parameter (that is, *k* does not have to be greater than 1), although it still does make the assumption that all the distributions are von Mises and all have a common concentration parameter (*k*).

Thus, it seems that the P-test test should be further investigated, especially with respect to its behaviour when its key assumptions are departed from. The test is defined as follows. It hinges on calculation of a *P*-statistic with is compared with the *F*-distribution with (*k*-1) and (*n*-*k*) degrees of freedom – where *k* is the number of samples, and n is the total number of data-points in all the samples combined.

The P-statistic is as follows:

$$P= \frac{\left( n-k \right)\left( \left( \sum_{i=1}^{k} R_{i} \right)-R \right)}{\left( k-1 \right)\left( n-\sum_{i=1}^{k} R_{i} \right)}+\frac{\left( n-k \right)\left( \tilde{R}-R_{bar} \right)\left( 1-R_{bar} \right)}{2\left( k-1 \right)\left( 1-\tilde{R} \right)}$$

Where *R_j_* is the resultant length of sample *j*, which *is sample mean resultant len*gth of sample *j* multiplied by the sample size of sample *j*.

*R* and *R_bar_* are the resultant length and sample mean resultant length of the combined set of all *n* data points, and thus *R* = *nR_bar_*.

And $\tilde{R}=\left( \frac{1}{n} \right)\sum_{i=1}^{k} R_{i}$

The sample mean resultant length is defined in section 3.2 of Pewsey et al. (2013).

**Rao’s homogeneity test on large samples (Rao polar test) and test for equality of dispersions (Rao dispersion test)**

The homogeneity test tests the null hypothesis that the underlying populations are identical. It requires the assumptions that the populations are unimodal and do not have means that are polar opposites, it also rests on the assumption that the sample sizes are sufficiently large that the test statistic can be compared to a chi-squared distribution with one degree of freedom – although how large this should be remains unexplored.

For sample *A* we define the following:

$$\begin{matrix} X_{a}=\frac{1}{n}\sum_{i=1}^{n} Cos\left( \alpha_{i} \right) & Y_{a}=\frac{1}{n}\sum_{i=1}^{n} sin\left( \alpha_{i} \right) & T_{a}=\frac{Y_{a}}{X_{a}} \end{matrix}$$

We define *X_b_*, *Y_b_* and *T_b_* similarly.

We then define

$$\begin{matrix} A_{ss}=\sum_{i=1}^{n} {Sin}^{2}\left( \alpha_{i} \right) & A_{cc}=\sum_{i=1}^{n} {Cos}^{2}\left( a_{i} \right) & A_{cs}=\sum_{i=1}^{n} Cos\left( \alpha_{i} \right)Sin\left( \alpha_{i} \right) \end{matrix}$$

We can define *B_ss_*, *B_cc_* and *B_cs_* similarly.

We define the asymptotic estimated variance associated with sample *A* as

$$a^{2}=\frac{1}{n}\left( \frac{A_{ss}}{X_{a}^{2}}+\frac{Y_{a}^{2}A_{cc}}{X_{a}^{4}}-\frac{2Y_{a}A_{sc}}{X_{a}^{3}} \right)$$

Again we define *b^2^* similarly. We can finally calculate the test statistic *H*

$$H=\frac{T_{a}^{2}}{a^{2}}+\frac{T_{b}^{2}}{b^{2}}-\frac{\left( \frac{T_{a}}{a^{2}}+\frac{T_{b}}{b^{2}} \right)^{2}}{\frac{1}{a^{2}}+\frac{1}{b^{2}}}$$

This test is implemented within Circstats, but its performance has not been explored.

The associated test for equality of dispersions makes the same assumptions but tests the null hypothesis that the two populations have the same dispersion. Again the test statistic is compared to a chi-squared distribution with one degree of freedom. Based on the quantities above we now define:

$$U_{a}=X_{a}^{2}+Y_{a}^{2}.$$

We define *U_b_* similarly. This is a measure of the concentration of the population, the associated asymptotic estimated variance associated with sample *A* is now given by

$$s_{a}^{2}=\frac{4}{n}\left( X_{a}^{2}A_{cc}+Y_{a}^{2}A_{ss}+2X_{a}Y_{a}A_{cs} \right)$$

We can define *s_b_^2^* similarly. This then allows us to define the test value:

$$H=\frac{U_{a}^{2}}{s_{a}^{2}}+\frac{U_{b}^{2}}{s_{b}^{2}}-\frac{\left( \frac{U_{a}}{s_{a}^{2}}+\frac{U_{b}}{s_{b}^{2}} \right)^{2}}{\frac{1}{s_{a}^{2}}+\frac{1}{s_{b}^{2}}}$$

Again, this test is implemented within Circstats, but its performance has not been explored.

**Circular ANOVA approaches**

We used three circular ANOVA approaches implemented in the R package *Directional* (Tsagris et al. 2020) and re-coded for faster performance in the package *Rfast* (Papadakis et al. 2019). The first one (Log-likelihood ratio ANOVA, function: lr.circaov) employs a log-likelihood ratio test, the second (Non equal concentration parameters approach ANOVA, function: het.circaov) uses the non-equal concentration parameters approach and the third (Embedding approach ANOVA, function: embed.circaov) uses an embedding approach to calculate significance.

**MANOVA approach**

This approach is based on the fact that every angle can be calculated by the summation of two orthogonal vectors, which constitute the sine and cosine of the angle. Therefore, each angle in the two samples can be split into two components by calculating its sine and cosine. Such a trigonometric approach has been previously described for linear models where sine(angle) and cosine(angle) were used as independent variables (Pewsey et al. 2013). However, if the goal is to compare two groups, sine(angle) and cosine(angle) would constitute response variables with a grouping variable as the independent factor identifying which sample the angle was derived from. This is done using a multivariate analysis of variance (MANOVA) generating a summary.manova object in R, using the default parameters (which employs the Pillai-Bartlett statistic). This generates a number of different p-values (derived from different approximations to the underlying *F*-statistic – but these p-values are all the same for the simple case of two groups discussed here, and can be interpreted as the p-value associate with the null hypothesis that the two samples are drawn from the same underlying population.

**References**

Batschelet E (1981) Circular statistics in biology. Academic Press, New York.

Fisher NI (1995) Statistical analysis of circular data. University Press, Cambridge, 296 pp.

Kuiper NH (1960) Tests concerning random points on a circle. In: Nederl. Akad. Wetensch. Proc. Ser. A. , 38–47.

Levene H (1960) Robust tests for equality of variances. In ‘Contributions to probability and statistics: essays in honor of Harold Hotelling’.(Eds I Olkin, SG Ghurye, W Hoeffding, WG Madow, HB Mann) pp. 278–292. Stanford University Press: Palo Alto, CA, USA.

Mardia KV (1967) A non-parametric test for the bivariate two-sample location problem. Journal of the Royal Statistical Society. Series B (Methodological) 29: 320–342.

Mardia KV, Jupp PE (2009) 494 Directional statistics. Wiley.

Paltani S (2004) Searching for periods in X-ray observations using Kuiper’s test-Application to the ROSAT PSPC archive. Astronomy & Astrophysics 420: 789–797.

Papadakis M, Tsagris M, Fafalios S, Dimitriadis M (2019) Rfast2: A Collection of Efficient and Extremely Fast R Functions II. Available from: https://CRAN.R-project.org/package=Rfast2.

Pewsey A, Neuhäuser M, Ruxton GD (2013) Circular statistics in R. Oxford University Press.

Rumcheva P, Presnell B (2017) An improved test of equality of mean directions for the Langevin-von Mises-Fisher distribution. Australian & New Zealand journal of statistics 59: 119–135.

Stephens MA (1972) Multisample tests for the von Mises distribution. Journal of the American Statistical Association 67: 456–461.

Tsagris M, Athineou G, Sajib A, Amson E, Waldstein MJ (2020) Directional: Directional Statistics.

Upton GJ (1976) More multisample tests for the von Mises distribution. Journal of the American Statistical Association 71: 675–678.

Watson GS (1962) Goodness-of-fit tests on a circle. II. Biometrika 49: 57–63.

Watson GS (1983) Statistics on spheres.

Watson GS, Williams EJ (1956) On the construction of significance tests on the circle and the sphere. Biometrika 43: 344–352.

Wheeler S, Watson GS (1964) A Distribution-Free Two-Sample Test on a Circle. Biometrika 51: 256. https://doi.org/10.2307/2334214

Zar J (2009) Biostatistical analysis. 5th edition. Prentice Hall, New York.
